# Benefit Finding and Related Factors of Patients with Early-Stage Cancer in China

**DOI:** 10.3390/ijerph19074284

**Published:** 2022-04-03

**Authors:** Xichenhui Qiu, Kefan Zhang, Yan Zhang, Liyuan Sun

**Affiliations:** Health Science Center, Shenzhen University, Shenzhen 518060, China; qiuxichenhui@szu.edu.cn (X.Q.); zhangkefan@szu.edu.cn (K.Z.); xiahzhang@szu.edu.cn (Y.Z.)

**Keywords:** benefit finding, early-stage cancer, social support, coping mode

## Abstract

(1) Background: Although the research on benefit finding (BF) in China has increased in recent years, it remains in its infancy. Few previous studies have focused on early-stage cancer patients. Therefore, this research study aimed to explore BF and its influencing factors for early-stage cancer patients in China. (2) Methods: From April to August 2019, 319 patients with early-stage cancer in the treatment period were selected by the convenience sampling method and evaluated using the Benefit Finding of Cancer Patients Scale-Chinese (BFS-C), Perceived Social Support Scale (PSSS), and Medical Coping Modes Questionnaire (MCMQ). (3) Results: The mean BF score was 47.57 (SD = 12.26). The results of the correlation analysis show that benefit finding was positively correlated with social support, but negatively correlated with acceptance-resignation. In addition, social support was negatively correlated with avoidance and acceptance-resignation. The results of the multiple linear regression indicate that the variables of self-assessment of disease severity, exercise time, coping mode (acceptance-resignation), and social support, affect BF. Finally, social support was shown to exert an intermediary effect on acceptance-resignation and BF. (4) Conclusions: In this study, the score of BF of patients with early-stage cancer was low. Medical staff should be more aware of the health behavior of patients with early-stage cancer, guide them to actively face the disease, and fully mobilize the social support of patients’ friends and family, so as to help patients increase their disease BF.

## 1. Introduction

China has the highest rate of cancer in the world. It was reported that there were 19.29 million new cancer cases worldwide in 2020, including 4.57 million new cancer cases in China, accounting for 23.7% of the global new cancer cases [1]. Cancer patients generally have different psychological problems, such as anxiety, irritability, fear, pessimism, etc. [2,3]. The emergence of these adverse psychological problems leads to the decline in the patients’ quality of life [4,5] and affects patients’ treatment compliance and satisfaction [6], in turn indirectly increasing medical expenses [7].

As one of the most influential research fields in positive psychology, benefit finding (BF) is becoming increasingly valuable among cancer patients. This term refers to a process of positive changes or benefits perceived by patients based on their illness experience, including personal, social, spiritual, and psychological cognitive and behavioral positive responses [8,9,10]. BF helps to improve endocrine and body immunity and affects healthy behavior and perceived social support, so as to promote the rehabilitation of disease [11,12]. Existing studies have shown that BF is related to demographic factors. Dunn’s [13] study of 439 cancer patients showed a higher level of benefit discovery among female patients. Tomich [14] found that economic status is related to BF. The average monthly income of the family is positively correlated with BF; that is, the higher one’s family income is, the greater the level of the BF [15]. Some studies have arrived at the opposite conclusion [16], observing that the lower the socioeconomic status of the individual is, the more benefit can be perceived from the disease. Lechner’s study [17] showed no link between income and BF.

Studies on disease-related factors and BF have shown that the later the pathological stage of cancer is, the lower BF will be [18]. Lechner’s study [19] found that under the same pathological stage, the patients’ perceived disease severity was significantly correlated with the score of BF. However, some scholars have stated the opposite [20], which may be related to specific diseases and different research objects. Gardner’s research [15] showed that multiple treatment and disease recurrence were negatively correlated with BF, indicating that the more the disease relapses, the lower BF will be. In addition, positive and effective coping styles can increase BF [21]. The reason for this may be that positive coping style can mobilize personal and social resources so as to actively and effectively deal with stressors, in turn promoting BF. Tougas [22] also confirmed that patients’ perception was that the higher the level of support from family and friends was, the stronger BF from the disease would be.

Although research on BF has been gradually carried out in China in recent years, the current research is still in the primary stage. These studies have mostly focused on breast cancer patients, and there have been few studies performed on early-stage cancer patients. At the same time, due to the differences between Chinese and Western culture, foreign research results may not be completely suitable for Chinese patients. Therefore, investigating the characteristics and influencing factors of BF of Chinese early-stage cancer patients will aid clinical medical staff in better determining the psychological adaptation of patients, so that they may take early intervention measures to promote the healthy behavior of patients and improve their quality of life.

## 2. Materials and Methods

### 2.1. Study Design

This cross-sectional study design was conducted to explore BF and its affecting factors on early-stage cancer among Chinese patients.

### 2.2. Setting and Samples

In this study, patients hospitalized in a third-grade class-A cancer hospital in China from April 2019 to August 2019 diagnosed with early-stage cancer and willing to participate in this study were selected as the research objects. The inclusion criteria were as follows: (1) age ≥ 18 years old; (2) primary school education and above; (3) patients with stage Ⅰ to Ⅱa (TNM stage) malignant tumors diagnosed by pathological examination; (4) patients with cancer diagnosed for 6 weeks to 1 year; and (5) patients with cancer after surgery or/and radiotherapy and chemotherapy, without significant progress of the disease at the time.

The exclusion criteria were as follows: (1) those who could not undergo investigation due to physiological, psychological, or emotional reasons; (2) people with communication difficulties or cognitive impairments; (3) patients with cancer metastasis or other history of cancer; and (4) people who were participating or had participated in other research studies.

**Figure 1:**
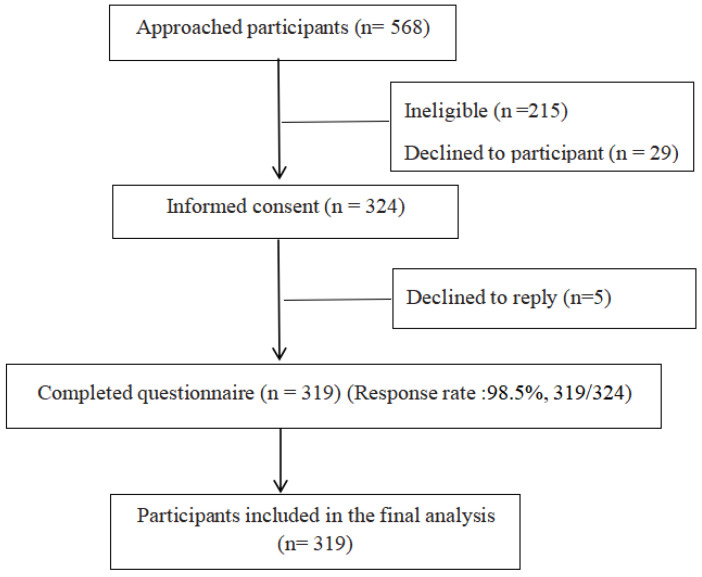
Recruitment and participant flow in this study.

According to the principle that the sample size in the multiple regression equation should be 10 times the number of independent variables [23], in this study the maximum number of independent variables that may affect BF was 27; thus, the appropriate sample size was 270. The study conservatively estimated that 20% of the questionnaires were invalid, and the final sample size was finally determined to be 324. A total of 319 valid questionnaires were collected in this study, for an effective recovery rate of 98.5%. See Figure 1 for details.

### 2.3. Measures

#### 2.3.1. General Information Questionnaire

This includes sociodemographic characteristics data (age, ethnic minority, gender, occupation, education level, marital status, economic income, personality type, medical security mode, etc.) and disease-related data (current main symptoms, self-perceived disease severity, daily exercise time, etc.).

#### 2.3.2. Benefit Finding of Cancer Patients Scale—Chinese (BFS-C)

The scale was adapted by Weaver et al. [24], then translated and localized by Chinese scholars [25]. The scale consists of the six dimensions of “acceptance”, “family relationship”, “personal growth”, “world outlook”, “social relationship”, and “healthy behavior”, with a total of 22 items. Patients were asked about each item in the following manner: “suffering from cancer (experience since diagnosis)...”. Likert grade 5 score was adopted, in which 1 point means “none at all” and 5 points means “very much.” The total score is the sum of the item scores, which is 22–110 points. The higher the score is, the stronger the disease BF will be. The Cronbach’s α coefficient of the scale of this study was 0.958.

#### 2.3.3. Perceived Social Support Scale (PSSS)

The scale was prepared by Blumenthal et al. [26]. It is used to measure the degree of support that individuals perceive from various social support sources, and the total score reflects the total degree of social support that individuals perceive. Chinese scholars introduced the scale and verified its good reliability and validity [27]. There are 12 items in the scale, and each item is scored according to level 1 to 7. In this study, the Cronbach’s σ coefficient of the perceived social support scale was 0.933.

#### 2.3.4. Medical Coping Modes Questionnaire (MCMQ)

The present study used this scale to assess the coping characteristics of early-stage cancer patients as a specific life event. The questionnaire was prepared by Feifel [28], and the Chinese version was translated and revised by Shen X et al. [29]. The questionnaire includes 20 items which measure three dimensions (coping modes). The three dimensions are confrontation, avoidance, and acceptance-resignation. “Confrontation” refers to problem coping, while “avoidance” and “acceptance-resignation” refer to emotional coping. Each item of the scale is scored according to level 1 to 4. The Cronbach’s σ coefficient of the scale in this study is 0.854.

### 2.4. Data Collection

The survey was conducted in the respiratory department, digestive department, gynecology department, head and neck tumor, and other wards of the inpatient department of the hospital. First, the clinical oncologist reviewed the medical records of the patients, obtained information regarding the disease diagnosis and pathological reports, and preliminarily selected the research subjects that met the inclusion criteria. Next, the researchers introduced the purpose and significance of the investigation to the patients, informed them of anonymity, confidentiality, and voluntariness, and obtained their consent by having them sign the informed consent form. After the patients had completed the relevant diagnosis and treatment measures on the same day, the researchers separately distributed the questionnaire to them and used unified guidelines to guide them in filling out the forms independently. The questionnaire was distributed and recovered on site.

### 2.5. Data Analysis

The data analysis was performed using the SPSS 26.0 software. Statistical significance was set at 0.05. Descriptive statistics were conducted to describe the sociodemographic characteristics of patients by means and standard deviations (SDs) and frequencies. Multiple linear regression analysis was used to explore the affecting factors of BF [23]. Pearson correlation analysis was applied to analyze the correlation among BF, social support, and coping style. Multiple linear regression was used to analyze the mediating role of social support between medical coping style and BF.

### 2.6. Ethical Considerations

This study was approved by the Research Ethics Committee of Health Science Centre, Shenzhen University. Before recruitment, a data sheet was distributed to all eligible patients to describe the purpose and process of the study. Prior to data collection, each participant gave their informed consent. Participants were informed that they could back out of the study at any time, and all collected data were processed anonymously and confidentially.

## 3. Results

### 3.1. BF and Score of Each Dimension

The mean score of BF of 319 patients was 47.57 (SD = 12.26), and the average score of each item was 2.56 (SD = 0.56). Among the six dimensions, the dimension with the lowest average score of items was “healthy behavior”, which was 2.05 (SD = 0.64). See Table 1 for details.

### 3.2. Sociodemographic Characteristics and Comparison of BF Scores of Patients with Different Characteristics

Among the respondents, 204 were female (63.9%) and 115 were male (36.1%). Middle-aged people aged 40–59 accounted for 48.6%, those with college degrees or above accounted for 38.5%, married people accounted for 86.5%, and those who had a per capita monthly income of less than CNY 10,000 (about US 1600) accounted for 73.1%. A total of 77.7% of the patients rated their current severity of the disease as moderate or above. The main accompanying symptoms of the disease were anorexia (34.2%), poor sleep (42.6%), and pain (32.0%). All of the variables were normally distributed at the overall level of BF. The results of the univariate analysis reveal that the three variables of education (F = 0.512, *p* = 0.045), self-rated disease severity (F = 4.076, *p* = 0.018), and daily exercise time (F = 6.904, *p* = 0.009) were statistically significant (Table 2).

### 3.3. Correlation Analysis of BF, Coping Style, and Social Support

The results of the Pearson correlation analysis (Table 3) show that BF was positively correlated with social support (r = 0.193, *p* < 0.05) and negatively correlated with acceptance-resignation (r = -0.160, *p* < 0.05). In addition, social support was negatively correlated with avoidance (r = 0.153, *p* < 0.05) and acceptance-resignation (r = 0.158, *p* < 0.05).

### 3.4. Analysis of Influencing Factors of BF

In order to further clarify the degree of impact of each variable on BF of the cancer patients, according to the univariate analysis result, the variables with statistically significant correlation or difference (*p* < 0.1) were selected as independent variables (see Table 4 for the assignment of independent variables), so as to perform multiple stepwise linear regression analysis. The results show that there were four variables affecting BF (*p* < 0.05), which could jointly explain 63.3% of the total variation. The results are shown in Table 5.

### 3.5. Mediating Role of Social Support

We assumed that social support was an intermediate variable between acceptance-resignation and BF, and the forced inclusion method was used for regression analysis. First, when regression analysis was carried out with BF as the dependent variable and acceptance-resignation as the independent variable for regression analysis, then *β* = −0.657 (*p* = 0.004) and *R* = 0.356. In addition, when using social support as the dependent variable and acceptance-resignation as the independent variable for regression analysis, then *β* = −0.392 (*p* = 0.005) and *R* = 0.441. Finally, when using BF as the dependent variable and acceptance-resignation and social support as the independent variables for regression analysis, then *β* = −0.413 (*p* < 0.001) and *R* = 0.316. These results show that when social support variables were added to the regression equation, then the absolute value of the regression coefficient of acceptance-resignation in BF decreased (from −0.657 to −0.413). This reveals that social support plays a partial mediating role in the prediction of the acceptance-resignation dimension of BF (see Table 6). 

## 4. Discussion

### 4.1. BF Level in Patients with Early-Stage Cancer

The results of this study show that the score of BF of patients with early-stage cancer was low (the average scores of each item were <3 and ≥3, which is the classification standard [30]), and these scores are lower than the results of domestic research [31]. Previous studies have shown that [32,33] the duration of illness is one of the factors affecting the BF of cancer patients. With the extension of the duration of illness, the level of BF of patients also gradually increases. The subjects of this study were patients who had been diagnosed with early-stage cancer for 6 weeks to 1 year. The short duration of illness may have been the reason for the low level of BF. In addition, some Chinese scholars have discussed the development track of BF in cancer patients from diagnosis to disease rehabilitation, and found that the development track of BF in patients includes a high-stability type, low-stability type, growth type, and decline type, thus indicating that the psychological characteristics of patients in various development tracks are different [34]. This study found that the BF level of early-stage cancer patients was low, but it cannot describe the movement track of BF. In the future, it is necessary to further adopt the research method of combined cross-sectional and longitudinal research to further explore the development track of the BF level and corresponding psychological characteristics.

This study also found that the average score of the dimension of healthy behavior was the lowest (2.05 ± 0.64), thus indicating that patients with early-stage cancer were not sufficiently active in improving their bad living habits, establishing a healthy diet and regular exercise, and having a healthier lifestyle. It is suggested that medical staff pay more attention to the health behavior of patients with early-stage cancer, guide them to eat and exercise healthily, and promote the development of a healthy lifestyle.

### 4.2. Influencing Factors of BF in Patients with Early-Stage Cancer

#### 4.2.1. Disease Severity

This study found that there was a positive correlation between self-perceived disease severity and BF, which is consistent with the results of other research studies [17]. Disease severity is an important factor affecting the BF of cancer patients [32,35]. If patients do not have medical knowledge, then their self-perceived disease severity will be different from the pathological stage of cancer. The subjective perceived disease severity of patients may beneficially affect their cognitive and behavioral changes in making some positive adaptive adjustments. A longitudinal study of early-stage breast cancer patients found that when the disease was diagnosed initially and after one year of illness, the perceived severity of illness was positively correlated with BF [36].

#### 4.2.2. Exercise Training

This study found that patients who exercised more than 1 h a day were positively correlated with BF. The reason for this may be that exercise is the main influencing factor in promoting patients’ social adaptation. The psychosocial adaptation level of patients who often exercise is also high [37]. Exercise can not only improve their physical function, but also improve their emotional state, enhance appetite, and alleviate clinical symptoms [38,39]. Patients also increase the time spent meditatively thinking during exercise, often reflect on themselves, and seek personal growth [40]. These findings suggest that medical staff should encourage cancer patients to engage in moderate physical exercise every day, strengthen physical function, and improve their psychological comfort and quality of life.

#### 4.2.3. Coping Style

The way of response refers to cognitive and behavioral approaches that individuals adopt under frustration and stress [28], and a positive one can act as a protective factor against psychological stress [41]. In this study, avoidance and acceptance-resignation were negatively correlated with BF, and in particular the patients who adopted acceptance-resignation had a greater impact on the BF. The reason for this may be that patients face great psychological pressure after being diagnosed with cancer and will show a negative emotion of letting go. Although acceptance-resignation can reduce the psychological pressure of patients in the short term [42], it will put patients in a negative mood in the long run, affect their initiative, and hinder the implementation of their health behavior, and is not conducive to patients’ perception of BF. It is thus suggested that medical staff strengthen the psychological guidance of patients and guide them in using positive coping styles to alleviate psychological stress.

#### 4.2.4. Social Support Is One of the Important Influencing Factors of BF

The results of this study show that there is a positive correlation between social support and BF. The higher the degree of social support is, the higher the level of BF will be. Other studies performed in China have confirmed that social support can improve the social adaptability of cancer patients and urge them to apply positive strategies to deal with problems [37,38,39,40]. Social support, as a powerful backup force when individuals encounter difficulties and setbacks, can enhance patients’ confidence in fighting diseases. The higher the perceived social support of patients is, the stronger their psychological adaptability will be, the lower the anxiety caused by the disease will be [43], and the more easily the patients will think about the benefits of the disease. At the same time, the results of multiple regression analysis show that social support exerted an intermediary effect on acceptance-resignation and BF; that is, acceptance-resignation had an impact on patients’ benefit finding through social support. Cancer is a stressor for any individual, and the internal support obtained by individuals under stress is insufficient. Therefore, the coping style of cancer patients requires social support to work effectively, so as to enhance disease BF. On the other hand, patients who tend to adopt negative coping will experience more negative outcomes because they will not positively seek additional support sources to deal with difficulties [44]. The results of this study suggest that medical staff should pay attention to guiding patients to actively face the disease, and fully mobilize the social support of patients’ friends and family to help patients increase their disease benefit-finding ability [45].

## 5. Conclusions

In this study, the BF level of early-stage cancer patients was shown to be low, and patients’ self-perception of disease severity, daily exercise time, social support, and coping style were observed to be the main factors affecting BF. Through the above factors, clinical medical workers can estimate the BF level of patients, identify patients with a low BF level as soon as possible, and take corresponding intervention measures. By providing high-quality humanistic care, clinical medical workers can encourage patients to actively face the disease and engage in appropriate physical exercise, guide patients’ families and society as a whole to offer the patients more support and care, and instruct patients to perceive the disease from a positive perspective, so as to improve their quality of life.

Due to the limitations of time and funding, this subject survey was only conducted via a cross-sectional survey from a time point after cancer patients fell ill; thus, it is not possible to grasp the dynamic change in BF over the course of the disease. In addition, due to the limitations of cross-sectional research, hybrid research methods must be adopted in the future to explore the relevant mechanisms and motion trajectories of BF in early-stage cancer patients, so as to better guide and serve clinical practice.

## Figures and Tables

**Table 1:** The mean scores of BF and its six dimensions.

Variable	Items	Mean ± SD (Scale/Dimension)	Mean ± SD (Item)
BF score (22–110)	22	47.57 ± 12.26	2.16 ± 0.56
Acceptance (3–15)	3	6.49 ± 1.9	2.16 ± 0.66
Family relationship (2–10)	2	4.23 ± 1.34	2.21 ± 0.67
World outlook (4–20)	4	9.06 ± 2.50	2.26 ± 0.62
Personal growth (7–35)	7	14.45 ± 4.81	2.21 ± 0.69
Social relationship (3–15)	3	6.48 ± 1.83	2.16 ± 0.61
Healthy behavior (3–15)	3	6.16 ± 1.93	2.05 ± 0.64

**Table 2:** Demographic characteristics of the study sample (N = 319).

Categorical Variables	N (%)	BF Scores	t/F	*p*
Sex			0.196	0.845
Male	115 (36.1)	47.39 ± 12.01		
Female	204 (63.9)	47.67 ± 14.43		
Age			0.937	0.334
18–39	88 (27.6)	45.76 ± 13.35		
40–59	155 (48.6)	47.96 ± 13.13		
60 and above	76 (23.8)	47.91 ± 11.51		
Ethnicity			0.004	0.952
Han	309 (96.9)	47.56 ± 12.29		
Other	10 (3.1)	47.80 ± 12.13		
Occupation			0.001	0.971
Worker	75 (23.5)	46.07 ± 11.56		
Military or civil servant	15 (4.7)	50.73 ± 10.15		
Professional technician	55 (17.2)	50.69 ± 12.79		
Business or service industry	60 (18.8)	45.78 ± 11.54		
Unemployed	57 (17.9)	48.72 ± 13.30		
Farmer	30 (9.4)	44.87 ± 11.58		
Other	28 (8.8)	48.11 ± 13.33		
Level of education			0.512	0.045
Primary school	39 (12.2)	47.03 ± 13.68		
Junior high school	71 (22.3)	46.68 ± 11.44		
High school	86 (27.0)	47.95 ± 11.37		
College degree or above	123 (38.5)	47.69 ± 13.42		
Marital status			1.053	0.297
Married	276 (86.5)	47.26 ± 12.06		
Single (unmarried, divorced, widowed)	43 (13.5)	49.56 ± 13.49		
Family per capita monthly income			0.389	0.533
CNY 5000 and below	123 (38.6)	47.08 ± 12.20		
CNY 5000–9999	110 (34.5)	48.14 ± 11.88		
CNY 10,000–14,999	44 (13.8)	49.84 ± 12.87		
CNY 15,000–19,999	12 (3.8)	49.42 ± 12.61		
CNY 20,000 and above	30 (9.4)	43.43 ± 12.56		
Form of medical security			2.497	0.115
Self-financed	33 (10.3)	44.76 ± 14.31		
Provincial or municipal medical Insurance	246 (77.1)	47.65 ± 11.92		
Other insurance	40 (12.6)	49.38 ± 12.47		
Are there any cancer patients in the family?			0.021	0.885
Yes	81 (25.4)	47.74 ± 11.98		
No	238 (74.6)	47.51 ± 12.38		
Main symptoms ^a^				
Chest tightness	54 (16.9)	46.96 ± 13.24	0.399	0.690
Anorexia	109 (34.2)	49.55 ± 12.05	2.088	0.068
Poor sleep	136 (42.6)	48.27 ± 12.50	0.880	0.379
Pain	102 (32.0)	49.45 ± 11.10	1.885	0.160
Diarrhea or constipation	78 (24.5)	48.12 ± 12.19	−0.451	0.652
Others	100 (31.3)	48.67 ± 12.58	−1.082	0.280
Combined with other diseases ^a^				
Hypertension	32 (10.0)	47.91 ± 12.06	−0.258	0.796
Diabetes	68 (21.3)	48.62 ± 11.08	−0.512	0.609
Heart disease	15 (4.7)	45.67 ± 13.54	0.615	0.539
Metabolic syndrome	11 (3.4)	50.36 ± 6.76	−0.768	0.443
Hyperlipidemia	18 (5.6)	42.72 ± 12.24	1.732	0.284
Chronic obstructive pulmonary disease	8 (2.5)	44.50 ± 4.54	0.717	0.474
Others	215 (67.4)	47.15 ± 13.00	0.883	0.378
Self-assessment of current disease severity			4.076	0.018
0–3	72 (22.3)	44.61 ± 12.61		
4–7	183 (57.4)	49.17 ± 12.30		
8–10	64 (20.3)	46.31 ± 11.13		
Personality self-assessment			1.266	0.261
Introverted	62 (19.4)	46.84 ± 12.85		
Extroverted	106 (33.2)	46.64 ± 11.44		
Ambivert	151 (47.4)	48.52 ± 12.59		
Exercise time per day			6.904	0.009
None	48 (15.0)	42.57 ± 11.09		
Less than 30 min	126 (39.5)	47.67 ± 11.40		
30 min to 1 h	103 (32.3)	48.33 ± 13.09		
More than 1 h	42 (13.2)	49.73 ± 12.00		
Therapeutic schedule			3.199	0.175
Operation	69 (21.6)	46.22 ± 49.99		
Chemotherapy	127 (39.8)	47.28 ± 11.28		
Radiotherapy	38 (11.9)	45.61 ± 12.36		
Two or more	85 (26.7)	49.99 ± 13.09		

Note: ^a^ refers to multiple options.

**Table 3:** Correlation analysis of BF, coping style, and social support (r).

Variables	Confrontation	Avoidance	Acceptance-Resignation	Social Support
Confrontation	-	-	-	-
Avoidance	0.043	-	-	-
Acceptance-resignation	0.087	−0.070	-	-
Social support	0.086	−0.153 *	−0.158 *	-
BF	0.008	−0.055	−0.160 *	0.193 *

* *p* < 0.05.

**Table 4:** Coding independent variables.

Independent Variables	Methods of Coding
Self-assessment of disease severity	* 0-3 = 1, 4-7 = 2, 8-10 = 3
Exercise time per day	* None = 1, Less than 30 min = 2, 30 min to 1 h = 3, More than 1 h = 4
Level of education	* Primary school = 1, Junior high school = 2, High school = 3, College degree or above = 4
Coping modes (Acceptance-resignation scores)	Original numerical value
Social support (PSSS scores)	Original numerical value

* Reference group.

**Table 5:** The multiple linear regression results of BF (N = 319).

Independent Variables	Unstandardized Coeffificients		Standardized Coeffificients (Ascending)	t	*p*
	B	Std. Error			
Constant	70.192	7.204	46.289	9.743	<0.001
Self-assessment of disease severity 2	3.566	1.662	3.566	2.146	0.033
Self-assessment of disease severity 3	3.099	2.042	3.099	2.763	0.009
Exercise time per day 4	5.175	2.549	5.175	2.030	0.043
Acceptance-resigna-tion	−1.157	0.503	−1.548	−2.298	0.022
Social support	0.148	0.054	1.879	2.724	0.007

F = 41.46, adjusted R^2^ = 0.633, *p* < 0.001.

**Table 6:** Verification of mediating effect of social support on acceptance-resignation dimension and BF.

Step	Dependent Variable	Independent Variable	*β*	*B* ^*^	*R*	*R* ^2^	*t*	*p*	*F*	*p*
1	BF	Acceptance-resignation	−0.657	−0.112	0.356	0.130	−3.239	0.004	8.479	0.004
2	Social support	Acceptance-resignation	−0.392	−0.158	0.441	0.127	−5.244	0.005	8.089	0.005
3	BF	Acceptance-resignation	−0.413	−0.063	0.316	0.184	−2.089	0.017	9.095	<0.001
		Social support	0.610	0.312			7.212	<0.001		

*: Standardized Coeffificients.

## Data Availability

The data presented in this study are available on request from the corresponding author. The data are not publicly available due to privacy restrictions.
